# Tackling antimicrobial resistance in practice: dental students’ evaluation of university teaching supplemented by an online course

**DOI:** 10.1093/jacamr/dlac039

**Published:** 2022-04-09

**Authors:** Lesley Cooper, Jacqueline Sneddon, Wendy Thompson, Tracey Guise, Douglas Robertson, Andrew Smith

**Affiliations:** 1 Scottish Antimicrobial Prescribing Group, Healthcare Improvement Scotland, Glasgow, UK; 2 British Society for Antimicrobial Chemotherapy, Birmingham, UK; 3 Division of Dentistry, University of Manchester, Manchester, UK; 4 Dental School, University of Glasgow, Glasgow, UK

## Abstract

**Background:**

Antimicrobial resistance (AMR) presents a global threat to public health. Engaging all healthcare professionals including undergraduates in efforts to tackle AMR is vital. Sharing and spreading good practice in teaching on AMR and antimicrobial stewardship (AMS) is a key ambition in Scotland. In 2020, the University of Glasgow Dental School supplemented teaching with mandatory completion by final year undergraduates of an online education programme on the essential role of dental teams in reducing AMR.

**Objectives:**

To evaluate final year dental students’ knowledge and experience of utilizing an online international educational, interactive resource to supplement university teaching: Tackling Antibiotic Resistance: What Should Dental Teams Do?

**Methods:**

Cross-sectional qualitative evaluation using a self-administered questionnaire with open questions about course content, learning and personal action planning. Data were thematically analysed using NVivo12 Pro software.

**Results:**

A total of 88 students completed a questionnaire, which indicated online training had increased their understanding of AMR and AMS from a global perspective and confirmed these topics were an integral part of their undergraduate education programme. Their action plans demonstrated enthusiasm for creating an AMS culture in clinical practice and an understanding of the need for ongoing education of themselves, their colleagues and patients.

**Conclusions:**

Education delivery using a variety of media to support teaching and learning in Glasgow Dental School was effective in ensuring that students understand their role in tackling AMR. Students were positive about the addition of an online education programme to supplement university teaching. This approach may be beneficial for other undergraduate dentistry programmes.

## Introduction

Antimicrobial resistance (AMR) is a global issue that represents a major threat to public health.^1^ AMR occurs naturally, however overprescribing and inappropriate use of antimicrobials accelerate the process. All healthcare professionals have a duty to ensure that they only prescribe antimicrobials when there are clear benefits for patients. Dentists are responsible for 7.2% of antimicrobial prescribing in primary care in Scotland,^2^ which is comparable with other parts of the UK and other high income countries.^3^ It has been suggested that as much as 80% of antibiotic prescribing by dentists may be inappropriate,^4,5^ therefore action to ensure effective antimicrobial stewardship (AMS) in this setting is important.^6^ Dental infections are readily amenable to procedures, such as tooth extraction, to remove the source of the infection and antibiotics are only usually necessary as an adjunct in the presence of systemic signs and symptoms of spreading infection. Antibiotics are inappropriate for the treatment of acute pain (toothache) from an inflamed pulp (pulpitis). Inappropriate antibiotic use by dentists is influenced by many factors and is highly dependent on context^5,7^. In the UK,^8^ inappropriate use often stems from avoiding or delaying dental procedures, whereas in the USA, overprescribing is more often related to prophylactic use. Giving antibiotics before an intervention to prevent a surgical site infection (for example, after wisdom tooth removal) or a distance site infection (such as infective endocarditis)^5^ are common examples.

Several studies from across the globe have concluded that despite an awareness among dentists and dental students that excessive use of antimicrobials leads to an increase in AMR, practice is not always consistent with local guidelines. Ongoing education is required to ensure optimal management of dental infections (which usually involves a procedure such as extractions or root canal treatment rather than prescription of antibiotics) and prophylactic prescribing in accordance with local guidelines.^3,9–12^ Studies have demonstrated that targeted education is an effective strategy to tackle AMR by improving dental prescribing, knowledge and confidence.^13,14^ Universities offering dental training are well positioned to promote positive behaviour in relation to AMS by integrating stewardship training in undergraduate programmes. AMS training should be offered using a variety of media including face-to-face education and self-directed online courses to meet the learning styles of learners.^15^

The Scottish Antimicrobial Prescribing Group (SAPG), part of Healthcare Improvement Scotland, established a Dental subgroup^16^ in 2018 to bring together work by national stakeholders, including dental schools and NHS Education for Scotland to support improved antimicrobial prescribing in management of dental conditions. Education was identified as a key area for further evaluation and development. Accordingly, to support good AMS by practitioners entering clinical practice, final year dentistry students (BDS5) at the University of Glasgow Dental School were mandated (in November–December 2020) to complete a free interactive online course organized by BSAC and the World Dental Federation (FDI) entitled ‘Tackling Antibiotic Resistance: What Should Dental Teams Do?’^17^ This course was launched in November 2020 to accompany the FDI white paper on the essential role of dental teams at tackling antibiotic resistance^6^ and has been undertaken by over 2000 learners from over 100 countries. This included some learners who are not clinical professionals such as prospective dental students. Course duration was 1 h per week for 3 weeks, undertaken at a convenient time each week, and supported by virtual discussion of issues with expert tutors and other learners. The course aims to increase awareness about antibiotic-resistant infections and show why they are a problem for everyone’s health and wellbeing. It aims to motivate behaviour change by sharing examples of how dental teams around the world have contributed towards tackling antibiotic resistance through preventing dental infections (for example through oral hygiene and diet advice), optimizing dental antibiotic prescribing (antibiotic stewardship) and raising awareness among patients about the risks of overusing antibiotics. The course also provides a framework for learners to create local solutions to help tackle the global problem of antibiotic resistance. FDI was keen to ensure relevance of the course for dental teams around the world. There are significant differences in the context and guidelines for dentistry around the world (including high-income and low- and middle-income countries) and in the local experience of antimicrobial stewardship. So this course provides a framework to encourage and support learners to identify issues and solutions relevant to their local context. This course is one of a series offered by BSAC in collaboration with FutureLearn providing free global access to high quality education^18^ on AMS and associated infection topics using a massive open online course (MOOC) model.

The aim of this study was to evaluate students’ perception of the value of the online course as an addition to undergraduate teaching. Associated objectives were to use a questionnaire to capture students’ feedback on the course and how they plan to use this learning to enhance stewardship behaviour in their future clinical practice. By sharing the results, we hope to inform AMR teaching approaches in other dental schools.

## Methods

### Teaching and learning provision

Within the Bachelor of Dental Surgery (BDS) course, teaching on dental antibiotic prescribing, resistance and stewardship is integrated within all modules involving infection delivered by academic staff (including dentists and researchers) throughout the 5 year course. This includes clinical teaching regarding treating dental infections with procedures rather than prescriptions wherever possible and infection-focused lectures and tutorials incorporating case studies delivered by a clinical oral microbiologist. Students undertake a series of formative assignments on a range of topics, including asking them to reflect on learning experiences. Their reflection on this MOOC, which focuses on how dentists in clinical practice can contribute to global efforts to tackle antibiotic resistance (as previously described) was undertaken as part of a formative assessment.

### Study design

This was a cross-sectional course evaluation study.

### Study population

Final year BDS students (*n *= 88) from the Dental School of the University of Glasgow were included.

### Intervention

**Figure 1.**
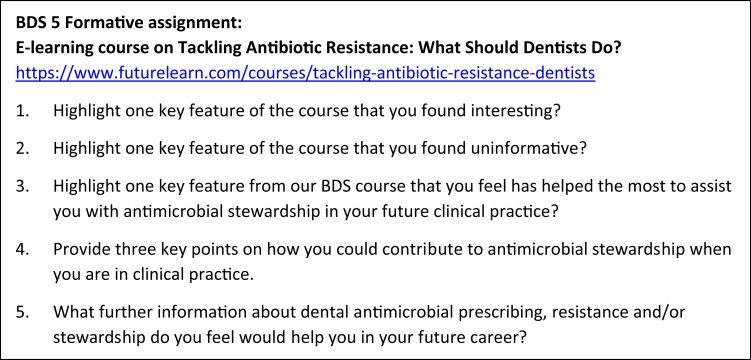
Questionnaire.

Based on similar questionnaires used for previous formative assessments with BDS5 students at University of Glasgow, a self-administered questionnaire (Figure 1) was created in a Word document and shared with students via e-mail. The three-part questionnaire asked students to highlight: one interesting feature of the online course; one uninformative aspect of it; and one key feature of the BDS course that had helped them the most with AMS in future clinical practice. Students were also asked to provide three points on how they could contribute to AMS in clinical practice. The open style of questions allowed students to give as much or as little detail as desired. Questionnaires were returned to A.S. who anonymized and depersonalized them before L.C. transferred information into NVivo 12 Pro software for analysis.

### Data analysis

Qualitative analysis was performed by one researcher (L.C.) using NVivo 12 Pro software and responses to each question were coded thematically according to the method described by Braun and Clarke.^19^

### Ethical approval

Ethical approval for this study was not required as data were collected as part of routine evaluation of education and all data were analysed and presented anonymously.

## Results

All 88 BDS5 students completed the course evaluation. Themes are presented according to responses to the separate questions, although crossover between some questions was observed. Overall students appeared to enjoy the course, thought it was well delivered and found the content engaging. The results are presented below in four sections, structured using the open-ended reflective questions asked of the students (see Figure 1): interesting features of the online course; uninformative aspects of it; utility of University of Glasgow’s BDS teaching on AMS for future practice; and the student’s plans for utilizing their learning in clinical practice. The subheadings within each section reflect the themes identified during analysis and highlight how many of the 88 students identified (unprompted) issues relating to that particular theme.

### Interesting features

#### Global differences

The most common feature (identified by 34 students) was the global variation in AMR and guidelines for antimicrobial agents to be used in treatment for dental infections. While most students who commented said the course showed how social and economic factors in each country made it necessary to adopt different approaches to AMS, a few felt there should be international consensus. Students particularly enjoyed hearing from international colleagues about their efforts in AMS and the inclusive nature of the interaction.

#### Increased understanding of AMR

That the course had increased their broader understanding of the development and spread of AMR and the adverse effects that can be associated with use of antibiotics was identified by 26 students. Aspects such as the contribution made by use of antibiotics in food production and animal health to the development and spread of resistance, WHO’s World Antimicrobial Awareness Week and its Access, Watch, Reserve (AWaRe) classification of antibiotics were new to some students as these were not covered in university teaching. Students also mentioned the need for a coordinated effort across different disciplines, sectors and countries to reduce unnecessary prescribing of antibiotics. Many students were surprised by the statistics regarding predictions of increased deaths resulting from AMR and identified these as both a cause for concern and a motivating factor to encourage clinicians to follow guidelines, participate in AMS and increase public awareness of AMR with the aim of slowing the prediction becoming a reality.

#### Patient experience

Some students specifically mentioned how invaluable they found the patient telling her own story about her life-changing experience of an infection that was resistant to antibiotics. They felt that it highlighted the reality of the situation and the pressing urgency to tackle AMR.

#### Course structure

Aspects of course presentation liked by the students included the videos and discussion sections, which made the course more interesting and interactive as well as giving the course structure. They felt that the quizzes at the end of each module (for example to select an Antibiotic Guardian pledge) were helpful to reinforce learning.

### Uninformative features

#### Repetitive

The most commonly reported ‘uninformative’ part of the course (identified by 22 students) was repetition, for example with respect to key messages about appropriate antibiotic use (stewardship). Some commented that this was because the information was covered earlier in the BDS undergraduate course whilst others thought that it was a good thing to reinforce important messages. One of the students recognized that course delegates from around the world were often saying and experiencing similar things.

#### Level of information

Some areas were felt to be basic, such as the impact of AMR on people. Students commented that further information on risk of *Clostridioides difficile* infection associated with antibiotic use would be useful, and so would information on how to discuss AMR with patients. Some students also reported the quizzes were too easy and could have been completed without completing course material beforehand.

#### Professional focus

A few students commented that the information could have been tailored more towards dentists or focused on UK issues and guidance. For example, a number of students commented that the content could have included the impact of COVID-19 on dental antibiotic use, including advice about managing patients with acute dental pain or infection in the new climate for dentistry. Some of the students shared their experience of increased patient requests (and in some cases demands) for antibiotics without a dental appointment (a practice not in accordance with clinical guidelines, as procedures are usually required instead of prescriptions).

### University of Glasgow BDS course content on AMS

#### Dental surgeons not dental prescribers

Thirty-nine students made comments that either directly or indirectly showed that AMS has been taught from the beginning and throughout several aspects of the BDS course including lectures, tutorials and in clinics. Eighteen students were very clear that they had been taught that treatment emphasis should be on dealing with the cause of infection rather than using antibiotics; many of these remarked they would take this with them into clinical practice. Nine out of 88 highlighted that immunology and microbiology courses had increased their understanding of AMR mechanisms and the need for stewardship. Eleven reported the clinical scenarios as particularly useful and that the interactive discursive nature of these sessions aided their learning.

#### Need to understand and adhere to prescribing guidelines

Twenty-three students discussed how elements of the BDS course had introduced and reinforced the need to practice according to antibiotic prescribing guidelines. Several students emphasized the need to keep up to date with guidelines as they may change over time.

### Future contribution to AMS—action plans

#### Create a culture of stewardship

Nearly three-quarters of the students (*n *= 65) identified the idea of providing AMR education to patients and/or colleagues Suggestions for how education should be conducted included: face-to-face conversations explaining the development and significance of AMR to patients and advising that antimicrobials are not always the correct treatment choice for dental and other infections, while ensuring they understand the importance of taking prescribed antibiotics as directed; educating all team members to ensure consistency of messages across the practice; videos, posters and leaflets in the waiting room; and a more aggressive approach to public awareness campaigning. Eighteen students discussed reviewing antibiotic use and learning with other members of the dental team to promote stewardship. Eight students would like education to support them to discuss AMR and reasons for not prescribing antibiotics with patients. Nineteen students identified regular auditing of antibiotic prescribing as important to monitor prescribing trends and evaluate practice.

#### Awareness of AMR and current guidelines for prudent prescribing

Nearly three-quarters of students (*n *= 65) reported wanting to keep up to date with current international, national and local guidelines on antimicrobial prescribing and emerging trends in AMR. Some students said awareness and easy access to local guidance on when to consider prescribing antibiotics and antibiotic choice would be helpful.

#### Local measures before prescribing

Forty-one students suggested first line of treatment for patients with toothache/abscess would be local measures such as incision and drainage or extirpation. Many showed awareness that these measures are the most effective treatment for symptom relief, therefore antibiotics should not be used as a ‘quick fix’ and only be used when necessary.

#### Prevention of dental infections

Twenty-seven students indicated that preventing and controlling infections was an important step in tackling resistance. They would encourage patients to have regular dental check-ups and focus on promoting good oral and dietary health, to reduce decay and infection, thereby reducing the need for antibiotics and decreasing AMR.

## Discussion

Undergraduate dental students valued learning about tackling antibiotic resistance from a combination of an online course and the more traditional teaching of their undergraduate training. The students valued many aspects of both learning opportunities and recognized their role in stewardship. Students reflected that some of the information within the MOOC had already been covered in the BDS course and suggested an online course tailored to their context would be more relevant to their learning needs. Students were positive about the online course format, supporting its utilization by other dental schools to supplement university teaching. This finding is important both for practice in the UK but also globally as in many countries AMS structures and processes may be less well developed with less strict controls on access to antibiotics.

The concept of MOOCs has changed since it was first described by Cormier in 2008.^20^ Initially it was envisaged as an open and continuous means of connecting students and tutors, beyond individual courses. Realizing that this was not a sustainable model, especially for individual tutors delivering many courses to a variety of students, the concept evolved until today it is synonymous with self-paced, online learning. The success of MOOCs^21^ as a concept is evidenced by the plethora of courses and several studies of their use in medical education have been reported. In a systematic review of published research about undergraduate medical education, Pei and Wu^21^ found no evidence that traditional (offline) lecture-based learning was better. In fact, there was evidence that online learning had advantages, including long-term retention of knowledge. For areas of specialist knowledge where there is a shortage of experts nationally, such as dental antimicrobial stewardship, MOOCs provide the advantage of sharing scarce resources.^22^ Together with the results of our study, a standardized approach to share course content across UK dental schools is advocated.

However, as also highlighted by the student feedback, MOOCs do have some problems. Content is out of the control of dental schools^22^ and keeping the content up to date is challenging in a rapidly changing environment, such as the COVID-19 pandemic. MOOCs are facilitated by peers rather than tutors. Furthermore, dental education is responsible for teaching ‘the attributes of professionalism’ and it is essential that dental schools continue to foster academic integrity and professionalism.^22^ As demonstrated in our study, a blended learning environment of both MOOCs and traditional formats may be the best of both worlds. Not all dental schools have the required expertise about antimicrobial prescribing, resistance and stewardship, so a MOOC provided by those with specialist expertise in dental AMS together with locally based university staff with professional expertise and enthusiasm for AMS can ensure it is woven throughout teaching over the 5 year degree course. The MOOC evaluated in this study, ‘Tackling Antibiotic Resistance: What Should Dental Teams Do?’^17^ could be utilized by other dental schools, although feedback from the students suggests that a more tailored version for undergraduates would be preferred and this could be updated more responsively.

To support patient-centred care and learn from patient narratives it is important to engage patients to support AMS, as illustrated by a toolkit for GPs that provides useful resources^23^ and a recent study on co-developing a shared decision-making AMS tool for use with dental patients.^24^

Students proposed actions for when they enter clinical practice that were positive with respect to AMS, demonstrating the impact that the combination of university teaching and the online course may have on attitudes and behaviours.^15,25^ This is important for the dental profession and broader dental teams and allows new practitioners to take the lead on AMS and champion good practice. Professional organizations also have a role in offering AMS courses for continuous professional development (CPD) post-registration.

### Conclusions

The online course entitled ‘Tackling Antibiotic Resistance: What Should Dental Teams Do?’ provided as a free resource by BSAC and FDI appears to make a valuable addition to university teaching for dental students and may have a positive impact on clinical practice around AMS for dentists entering the profession. We suggest that utilization of online learning such as that provided by this course should be considered by dental schools globally to support the contribution of dental teams to tackling AMR.

Further work would be required to explore the feasibility and impact of more dental schools engaging with this course since at present it has a diverse international audience and involves learner engagement via a facilitated discussion, which could potentially be overwhelmed if there was a large increase in learners.
